# Supplementary material for: Integrative study of pulmonary microbiome and clinical diagnosis in pulmonary tuberculosis patients
Source: Microbiol Spectr. 2025 Jun 20;13(8):e01563-24. doi: 10.1128/spectrum.01563-24 (PMC12323596; doi:10.1128/spectrum.01563-24)
Supplement: Supplemental figures — Figures S1 to S3. [file spectrum.01563-24-s0001.docx]

Fig S1. Detection of different methods in patients with confirmed TB


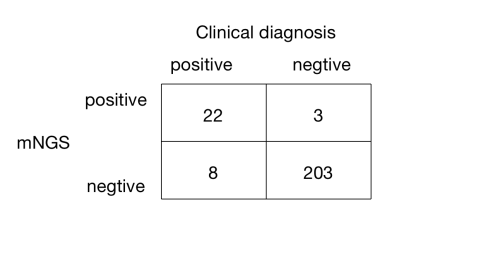

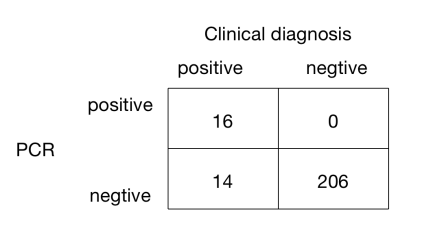

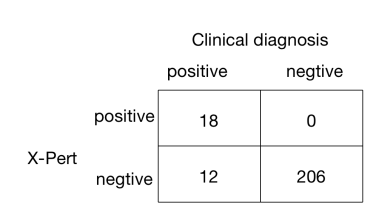


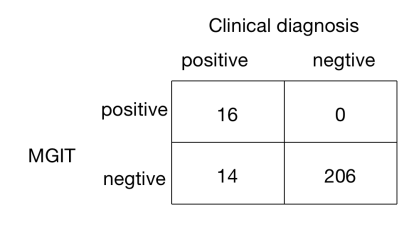

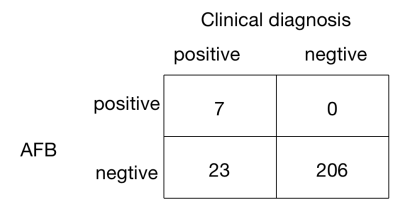

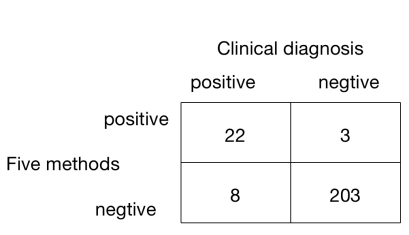


Fig S2. Diagnostic results of different methods for diagnosis of tuberculous.

Fig S3. advantage species among three groups
